# Sex as a moderator in the associations between psychopathy facets and aggressiveness

**DOI:** 10.3389/fpsyg.2025.1534317

**Published:** 2025-02-25

**Authors:** Sophie L. Kjærvik, Nicholas D. Thomson

**Affiliations:** ^1^Department of Surgery, Virginia Commonwealth University, Richmond, VA, United States; ^2^Department of Psychology, Virginia Commonwealth University, Richmond, VA, United States

**Keywords:** psychopathy, aggression, anger, hostility, sex difference

## Abstract

**Introduction:**

Psychopathy reliably predicts aggression, making it valuable for violence prevention. However, research on sex differences within the 4-facet model, which includes affective, interpersonal, lifestyle, and antisocial facets of psychopathy, is limited, especially among high-risk community samples.

**Methods:**

This study examined sex differences in the psychopathy facets associated with anger, hostility, and aggression among 419 (*M_age_* = 32.6, 72% male) violently injured adults. Studying high-risk, non-institutionalized individuals offers unique insights into the link between psychopathy and aggression, particularly in real-word context where institutional influences are absent. Participants completed the Self-Report Psychopathy and Aggression Questionnaire.

**Results:**

Hierarchical multiple regressions revealed that all four facets predicted physical and proactive aggression; affective, lifestyle, and antisocial facets were related to reactive aggression; and affective and lifestyle facets were related to anger, hostility, and verbal aggression. Sex moderated relations between psychopathy facets and anger and hostility. Specifically, the affective facet was associated with anger and hostility for males but not for females. The lifestyle facet was associated with anger and hostility for males and females, but the effect was stronger for females.

**Discussion:**

The findings indicate that the four-facet model relates to aggressive emotions and cognition differently for males and females, while demonstrating consistency in physical and verbal aggression. Recognizing that psychopathic anger and hostility are sex-specific can improve violence interventions tailored to males and females.

## Introduction

Psychopathy is characterized by a constellation of personal and behavioral traits, including lack of emotional depth, manipulativeness, arrogance, irresponsibility, and persistent antisocial behavior (Blair et al., 2005). Although the prevalence of psychopathy is relatively low (1–2% for males and 0.3–0.7% for females), individuals with psychopathic traits are estimated to account for 20–40% of violent crimes (Thomson and Kjærvik, 2024). Moreover, approximately 15–25% of prisoners are classified as psychopaths (Blair et al., 2005), and about 93% of individuals with psychopathic traits are involved in the criminal justice system, making it one of the costliest psychiatric disorders (Kiehl and Hoffman, 2011). Thus, understanding the relationship between psychopathy and aggression has become a critical focus of research. Unfortunately, most existing studies on psychopathy and aggression do not consider sex differences (Thomson, 2020), leaving a gap in our understanding of whether facets of psychopathy present as different risk factors for physical and verbal aggression in males and females. Additionally, limited research has examined the relation between psychopathy facets and anger and hostility, and no research has investigated if that varies by sex. This study aims to fill these gaps by examining whether the psychopathy facets are differently related to these aspects of aggressiveness among males and females.

### The four facets of Psychopathy

Psychopathy was originally considered as consistent of two factors (Hare et al., 1990), with Factor 1 assessing the core of psychopathy (e.g., shallow affect, lack of empathy, and manipulativeness), while Factor 2 assessed behavioral and criminal tendencies. The factor structure has been useful in predicting aggression, but studies with females show mixed results (de Vogel and de Ruiter, 2005; Salekin et al., 1997). Research has found that a four-facet model best captures the complexity of psychopathic traits (Neumann et al., 2007). The four facets are affective (e.g., lack of empathy, remorse, shallow emotionality), interpersonal (e.g., pathological lying, manipulative behaviors), lifestyle (e.g., stimulus seeking, impulsivity, erratic lifestyle), and antisocial (e.g., versatile antisocial behavior, poor behavior control). Although some argue against the inclusion of antisocial behavior in the construct of psychopathy (Skeem and Cooke, 2010), the four-facet model has proven beneficial for violence risk models. The antisocial facet controls for past and present crime; thus, the links between other facets of psychopathy and aggression are independent of criminality. Additionally, research with the four facets has shown that addressing the separate dimensions of psychopathy expands the understanding of the complex and often differential link between psychopathy and other variables that are important to the study of psychopathy (West et al., 2023). The four-facet structure has been validated in both female and male samples from over 58 nations (Neumann et al., 2012), and scholars argue that it provides a good understanding of sex differences in aggressive and violent behavior (Thomson, 2018).

### Theoretical foundation for sex differences in psychopathy and aggressiveness

The Social Role Theory posits that men are more aggressive due to social expectations that encourage dominance and competition in men and compliance and non-competitiveness in women (Wood and Eagly, 2012). Additionally, physical differences between men and women impact the development of aggression. Females are less likely to engage in overt aggression to reduce the risk of injury (Wood and Eagly, 2012), while males are more physically aggressive due to their larger and stronger bodies (Björkqvist, 2018; Vaillancourt, 2013). However, psychopathy has a masculine nature (Lyons et al., 2015), and females with psychopathic traits may be more likely to use overt forms of aggression than others. Indeed, females with affective psychopathic traits are more likely to use physical aggression (Thomson et al., 2019b). In contrast, prior research has found no difference in hostility and anger in females and males (Milovchevich et al., 2001). However, some argue that females with psychopathic traits are more impulsive, manipulative, and emotionally unstable than males (Forouzan and Cooke, 2005; Kreis and Cooke, 2012), indicating a possible sex difference in how psychopathy facets relate to anger and hostility. Regardless, empirical evidence on how psychopathy may differ in females compared to males is still scarce and inconsistent (Pinheiro et al., 2022, 2024).

### Psychopathy and anger

Anger is “an emotional response to a real or imagined threat or provocation” (Baumeister and Bushman, 2020, p. 201). While anger is often related to aggression, surprisingly little research has explored the connection between psychopathy and anger (Jackson et al., 2007). One reason may be the assumption that psychopathy is associated with reduced ability to experience negative emotions such as fear and sadness (Patrick et al., 1993), leading some to suggest a similar deficiency in experiencing anger (Cleckley, 1951). While others argue that psychopathy is associated with normal anger processing (Blair et al., 1997; Blair et al., 2005).

Further complicating this debate, some propose that only antisocial and lifestyle facets relate to anger because anger is linked to externalizing psychopathology and, thus, not the core of psychopathy (Fowles and Dindo, 2009; Hicks and Patrick, 2006). However, the development of psychopathic traits may involve learning to disregard emotions like sadness and fear while using anger in interpersonal situations to manage negative emotions (Kosson et al., 2020). Evidence supports this, showing that individuals with high psychopathic traits tend to be angrier (Coccaro et al., 2014) and exhibit chronic anger across various populations, including community participants, adult offenders, and detained adolescents (Kosson et al., 2020). Yet, which psychopathy facets contribute to anger remains unclear. In one study, only the lifestyle facet was related to anger (Hall et al., 2004). While in another study, the interpersonal and affective facets were related to anger (Zolondek et al., 2006), which challenges the argument that anger is unrelated to the core of psychopathy.

### Psychopathy and hostility

In contrast to anger, hostility is the cognitive component of aggression, characterized by a negative outlook toward others (Kjærvik and Bushman, 2024). The tendency to see ambiguous situations as hostile has been linked to psychopathy, particularly with lifestyle and antisocial traits. This aligns with the Social Information Processing theory, which suggests that individuals interpret social cues through their personal biases and beliefs, leading to diverse and faulty interpretations in ambiguous situations, sometimes resulting in hostile attributions (Dodge, 1980). Psychopathy is associated with difficulties in attending to subtle social cues, which can contribute to misunderstanding ambiguous or hostile situations (Law and Falkenbach, 2018; Maccoon and Newman, 2006). Indeed, individuals with psychopathic traits have stronger and less accurate hostile beliefs (Buades-Rotger et al., 2023). Research with prisoners has shown that all aspects of psychopathy are related to hostile attributes (Vitale et al., 2005). However, studies in community settings suggest that only lifestyle and antisocial traits relate to hostility (Law and Falkenbach, 2018), suggesting that the lifestyle and antisocial traits may relate to hostility in this sample.

### Psychopathy and aggressive behavior

Researchers distinguish between different types of aggression, including physical (e.g., hitting, kicking) and verbal aggression (e.g., arguing, insults; Anderson and Bushman, 2002). Psychopathy facets have been linked to distinct types of aggression across males and females. Among males, antisocial and lifestyle facets were associated with physical aggression and violence (Olver et al., 2013; Zwets et al., 2015). Among females, the antisocial and affective facets predicted future violence (Thomson and Kjærvik, 2024) and proactive aggression, while the lifestyle facet predicted reactive aggression (Thomson et al., 2019a). These findings show the need for more studies on whether psychopathy facets differently relate to aggressiveness in males and females, particularly as the four-facet model appears sensitive to these differences (Thomson et al., 2019a).

### The current study

This study aimed to address gaps in the literature by examining whether the four facets of psychopathy (affective, interpersonal, lifestyle, and antisocial) were differentially associated with anger, hostility, and physical and verbal aggression, and whether these associations varied by sex. Given the existing evidence that psychopathy manifests differently across males and females, this study tests the moderating role of sex in these relations. The antisocial facet was expected to relate to physical aggression across sexes. While the affective facet was expected to relate to physical aggression in females but not males. The interpersonal and antisocial facets were expected to relate to verbal aggression. The antisocial facet was expected to relate to verbal aggression in females but not males. The affective and lifestyle facets were expected to relate to anger. The moderating role of sex was explored, with the expectation that the link between these traits and anger would be stronger in females than males. The affective and lifestyle facets were expected to relate to hostility. The moderating role of sex was explored, with the expectation that the link between these traits and hostility would be stronger in females than males. By testing sex as a moderator, this study contributes to an improved understanding of the association between psychopathy and different dimensions of aggressiveness.

## Methods

### Participants

A total of 419 violently injured adults were included in this study. Participants ranged in age from 18 to 75 (*M* = 32.64, *SD* = 12.88), and the majority were biologically male (72%). Participants identified as Black/African-American (78%), White (16%), or other (6%; Asian, Native American, or mixed race). Participants were admitted to the hospital for sustaining gunshot wounds (53%), assault wounds (39%), or stab wounds (8%).

### Procedure

Adult patients who sustained violent injuries were identified and screened daily at a Level 1 Trauma Center in Virginia, with recruited occurring both in the emergency department and inpatient setting. To be eligible for the study, participants needed to have sustained a violence-related injury and be medically stable at the time of study participation. Individuals were excluded if they were either prisoners or minors under 18 years of age. The study specifically focused on individuals who were violently injured, recognizing that this group faces a high risk of subsequent violence and related injuries (Carter et al., 2018; Cunningham et al., 2009). Studying violently injured patients, who are at high risk for future violence but are not confined to institutional settings, offers a unique opportunity to understand these associations in a community context. Initial eligibility was determined using live medical records, followed by in-person confirmation. The study achieved a 75% recruitment rate. Before giving consent, participants were informed that their involvement was solely for research purposes and would not affect their medical care. At baseline assessment, participants engaged in semi-structured interviews and completed self-report questionnaires, which took approximately 2 h. They were compensated $160 for their time. The study was approved by the authors’ institutional review board and was granted a Certificate of Confidentiality by the CDC.

### Measures

#### Psychopathy facets

The Self-Report Psychopathy Scale-Short Form (SRP-SF) (Paulhus et al., 2012) is designed to measure psychopathic traits in non-clinical populations. The scale contains 29 items rated on a 5-point Likert scale (1 = strongly disagree, 5 = strongly agree). The scale measures psychopathy across four facets: affect (e.g., “I do not bother to keep in touch with my family any more”), interpersonal (e.g., “A lot of people are ‘suckers’ and can easily be fooled”), lifestyle (e.g., “I admit that I often ‘mouth off’ without thinking”), and antisocial (e.g., “I was convicted of a serious crime”). Scores from each dimension were sum-scored to create subscales. The measure is widely used in both research and applied settings for assessing psychopathic traits efficiently while maintaining strong psychometric properties (Mahmut et al., 2008).

#### Anger, hostility, physical, and verbal aggression

The Aggression Questionnaire (AQ) (Buss and Perry, 1992) consists of 29 items rated on a 5-point scale (1 = extremely uncharacteristic of me, 5 = extremely characteristic of me). The measure is widely used to assess anger (7 items), hostility (8 items), physical (9 items), and verbal (5 items) aggression. Subscales were sum-scored and showed good internal consistency (anger, *α* = 0.86; hostility, *α* = 0.88; physical, *α* = 0.78; verbal, *α* = 0.80). The measure has good psychometric properties and is commonly used in clinical and research settings to assess aggression (Bryant and Smith, 2001).

### Data analysis plan

Statistical analyses were conducted in R, chosen for its advanced capability in handling hierarchical regression models and flexible visualization tools (R Core Team, 2024). Hierarchical multivariate multiple regressions assessed the link between psychopathy facets and four aspects of aggression. Step 1 included sex, age, and the four facets, while Step 2 added the interaction terms between sex and each facet. Significant interactions were probed using simple slope analysis (Aiken and West, 1991). A prior power analysis with G*Power 3.1.9.6 for linear multiple regression with R^2^ increase, with *α* = 0.05, a power = 0.80, and an effect size = 0.10 indicated a sample size of 159 for the moderation analysis. To handle missing data, the mean of the respective Self-Report Psychopathy Scale or Aggression Questionnaire subscale was imputed, a widely used approach for addressing missing items (Segerstrom, 2020). This method was applied only to rows with fewer than two missing items, ensuring that no participants were excluded. Data from this study are not publicly available due to the sensitive nature of the population.

## Results

### Correlations between main study variables

Correlations are displayed in Table 1. All facets were positively correlated with all aspects of aggression (*ps* < 0.001). Sex was negatively related to affective (*p* = 0.03), interpersonal (*p* = 0.02), lifestyle (*p* = 0.02), and antisocial facets (*p* = 0.0002). Physical aggression was negatively related to sex (*p* = 0.03) and age (*p* = 0.03).

**Table 1:** Correlations between study variables.

	*M*	*SD*	1	2	4	5	6	7	8	9	10
Age	32.64	12.88	–								
Sex	0.28	0.45	−0.06	–							
Physical Agg.	23.90	7.58	−0.11*	−0.11*	–						
Verbal Agg.	13.90	5.23	−0.05	−0.007	0.59*	–					
Anger	13.72	6.39	−0.05	0.006	0.68*	0.65*	–				
Hostility	20.28	8.72	−0.003	−0.009	0.56*	0.59*	0.73*	–			
Affective	15.18	5.88	−0.09	−0.11*	0.56*	0.54*	0.57*	0.54*	–		
Interpersonal	12.09	5.24	−0.003	−0.14*	0.41*	0.38*	0.45*	0.42*	0.68*	–	
Lifestyle	14.16	5.82	−0.04	−0.11*	0.53*	0.51*	0.58*	0.53*	0.72*	0.69*	–
Antisocial	12.15	5.16	0.03	−0.18*	0.48*	0.39*	0.47*	0.37*	0.64*	0.70*	0.69*

*p < 0.05. Sex = (0) Male, (1) Female.

### Psychopathy facets and aggressiveness

**Figure 1:**
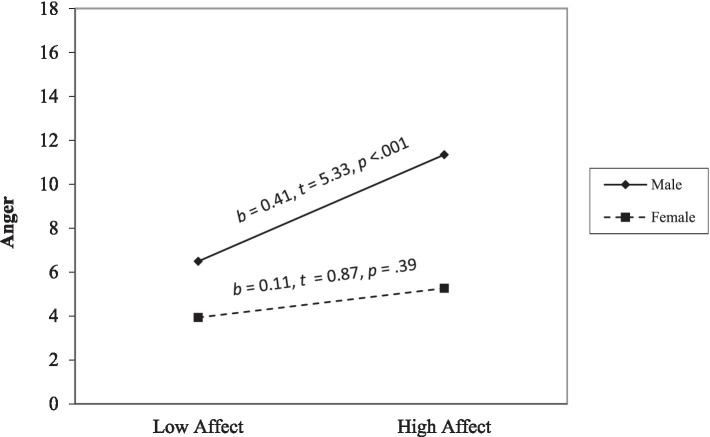
The moderating effect of sex on the link between the affect facet and anger. The low and high of the predictor represent ±1.0 *SD* from the mean. The low and high values of the moderator are 0 and 1.

**Figure 2:**
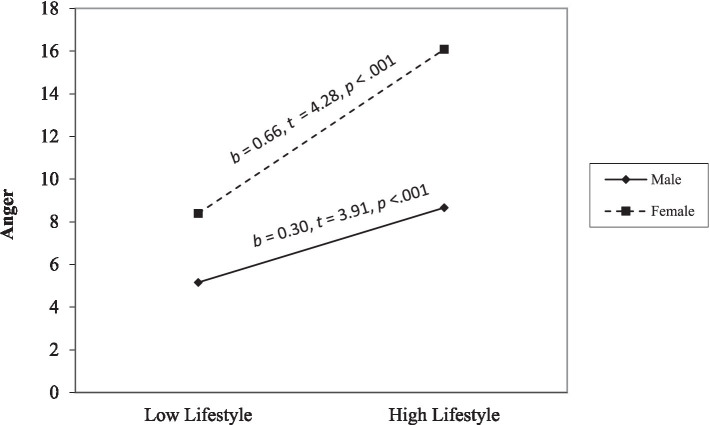
The moderating effect of sex on the link between the lifestyle facet and anger. The low and high of the predictor represent ±1.0 *SD* from the mean. The low and high values of the moderator are 0 and 1.

Hierarchical regressions are displayed in (Table 2). Anger. Step 1 was significant, *F*(6, 412) = 44.18, *p* < 0.001, and affective and lifestyle facets positively related to anger (*ps* < 0.001). Step 2 was significant, *F*(10, 408) = 27.71, *p* < 0.001. Affective and lifestyle facets positively related to anger (*ps* < 0.001). The interaction terms between sex and affective traits (*p* = 0.049) and sex and lifestyle traits were significant (*p* = 0.037). Simple slope analyses (see Figure 1) revealed that high affective traits were associated with more anger for males (*p* < 0.001) but not for females (*p* = 0.39). In contrast, high lifestyle traits were associated with more anger for males and females (*ps* < 0.001); however, the slope was less steep for males than females (see Figure 2).

**Table 2:** Hierarchical linear regression: psychopathy and sex as predictors of physical aggression, verbal aggression, anger, and hostility.

	Physical aggression				Verbal aggression				Anger				Hostility			
	*B*	SE	β	*R^2^*	*B*	SE	β	*R^2^*	*B*	SE	β	*R^2^*	*B*	SE	β	*R^2^*
Step 1				0.36***				0.32***				0.38***				0.33***
Age	−0.04	0.02	−0.07		−0.01	0.02	−0.002		−0.001	0.02	−0.003		0.03	0.03	0.05	
Sex	−0.54	0.67	−0.03		0.71	0.48	0.06		1.11	0.55	0.08*		1.15	0.78	0.06	
Affective	0.45	0.08	0.35***		0.35	0.06	0.39***		0.34	0.07	0.31***		0.56	0.09	0.37***	
Interpersonal	−0.18	0.09	−0.13*		−0.09	0.06	−0.09		−0.05	0.07	−0.04		0.05	0.11	0.03	
Lifestyle	0.32	0.08	0.24***		0.26	0.06	0.30***		0.38	0.07	0.34***		0.47	0.10	0.31***	
Antisocial	0.27	0.09	0.18**		0.01	0.06	0.01		0.08	0.07	0.07		−0.16	0.11	−0.10	
Step 2				0.36***				0.32***				0.39***				0.36***
Age	−0.04	0.02	−0.07		0.0001	0.02	0.0003		0.003	0.02	0.005		0.04	0.03	0.06	
Sex	1.06	1.93	0.06		2.11	1.38	0.18		0.23	1.59	0.02		0.37	2.23	0.02	
Affective	0.47	0.09	0.36***		0.37	0.06	0.41***		0.41	0.07	0.38***		0.71	0.10	0.48***	
Interpersonal	−0.25	0.10	−0.17*		−0.10	0.07	−0.10		−0.10	0.08	−0.08		0.03	0.12	0.02	
Lifestyle	0.32	0.09	0.24***		0.26	0.07	0.29***		0.30	0.08	0.27***		0.27	0.11	0.18*	
Antisocial	0.33	0.10	0.22***		0.03	0.07	0.03		0.11	0.08	0.09		−0.12	0.11	−0.07	
Sex*Affective	−0.08	0.18	−0.08		−0.08	0.13	−0.11		−0.30	0.15	−0.33*		−0.63	0.21	−0.51**	
Sex*Interpersonal	0.44	0.24	0.33^†^		0.06	0.17	0.07		0.25	0.20	0.23		−0.01	0.28	−0.008	
Sex*Lifestyle	−0.05	0.21	−0.04		0.006	0.15	0.007		0.36	0.17	0.37*		0.96	0.24	0.72***	
Sex*Antisocial	−0.42	0.24	−0.31^†^		−0.09	0.17	−0.10		−0.22	0.20	−0.19		−0.25	0.28	−0.16	

*p < 0.05; **p < 0.01; ***p < 0.001; ^†^*p* < 0.08. Sex = (0) Male, (1) Female.

**Figure 3:**
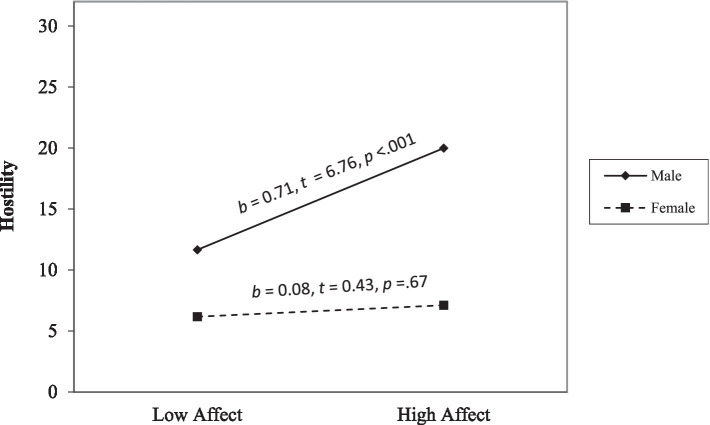
The moderating effect of sex on the link between the affect facet and hostility. The low and high of the predictor represent ±1.0 *SD* from the mean. The low and high values of the moderator are 0 and 1.

**Figure 4:**
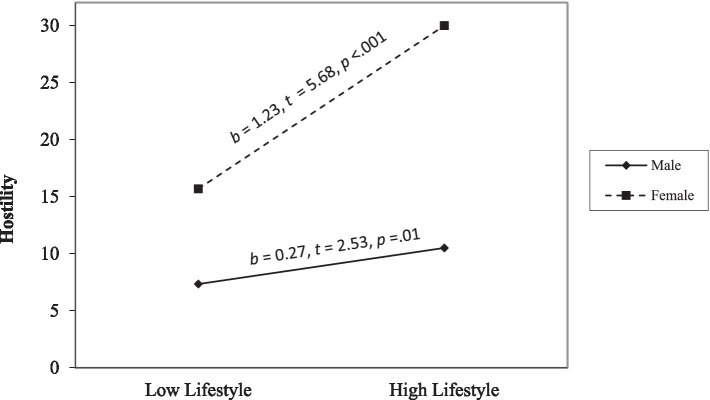
The moderating effect of sex on the link between the lifestyle facet and hostility. The low and high of the predictor represent ±1.0 *SD* from the mean. The low and high values of the moderator are 0 and 1.

Hostility. Step 1 was significant, *F*(6, 412) = 35.97, *p* < 0.001, and affective and lifestyle facets positively related to hostility (*ps* < 0.001). Step 2 was significant, *F*(10, 408) = 24.23, *p* < 0.001. Affective and lifestyle facets positively related to hostility (*p* < 0.001, *p* = 0.01). The interaction terms between sex and affective traits (*p* = 0.003) and sex and lifestyle traits were significant (*p* < 0.001). Simple slope analyses (Figure 3) revealed that high affective traits were associated with more hostility for males (*p* < 0.001) but not for females (*p* = 0.67). In contrast, high lifestyle traits were associated with more hostility for both males (*p* = 0.01) and females (*p* < 0.001); however, the slope was less steep for males than females (Figure 4).

Physical Aggression. Step 1 was significant, *F*(6, 412) = 40.24, *p* < 0.001. All four facets positively related to physical aggression (affect, *p* < 0.001; interspersion, *p* = 0.04; lifestyle, *p* < 0.001; antisocial, *p* = 0.003).^1^ Step 2 was also significant, *F*(10, 408) = 24.66, *p* < 0.001. All four facets positively related to physical aggression (affect, *p* < 0.001; interspersion, *p* = 0.01; lifestyle, *p* < 0.001; antisocial, *p* < 0.001). Interaction terms were non-significant.

Verbal Aggression. Step 1 was significant, *F*(6, 412) = 34.17, *p* < 0.001, and affective and lifestyle facets positively related to verbal aggression (*ps* < 0.001). Step 2 was significant, *F*(10, 408) = 20.52, *p* < 0.001, and affective and lifestyle facets positively related to verbal aggression (*ps* < 0.001). Interaction terms were non-significant.

## Discussion

No prior literature has examined sex differences in the link between psychopathy facets and anger and hostility. Additionally, there is scarce research on sex differences in the 4-facet model of psychopathy in relationship to aggressive behavior. To fill these gaps, this study examined sex differences in the associations between psychopathy facets and anger, hostility, and physical and verbal aggression. The results indicate that psychopathy facets are related to angry feelings, angry cognition, and aggressive behaviors. Notably, variations were observed across facets, showing that the four-facet model of psychopathy provides a deeper understanding of how psychopathy differs as a risk factor for these aspects of aggressiveness in males and females.

### Anger and hostility

Findings reveal sex-specific pathways linking affective and lifestyle facets to anger and hostility. The affective facet was related to anger and hostility in males but not females, suggesting it plays a crucial role in male anger. In contrast, the lifestyle facet related to anger and hostility in both sexes, with stronger effects in females. This aligns with prior research, suggesting that males with affective and males and females with lifestyle traits use anger to manage negative emotions to anger (Kosson et al., 2020; Velotti et al., 2024).

For males, the emotional deficits associated with affective traits may lead to increased anger due to emotional dysregulation and reduced empathy, potentially creating a hostile attribution bias (Law and Falkenbach, 2018). The lifestyle facet’s link to hostility, stronger in females, extends prior work by suggesting that the lifestyle facet, rather than the lifestyle-antisocial factor, drives hostile attribution bias (Law and Falkenbach, 2018).

Females with higher lifestyle scores may experience greater anger and hostility due to impulsivity, frustration from interpersonal conflicts, and societal consequences like social isolation. The impulsive, reckless behavior associated with this facet may create consistent patterns of negative social interactions, reinforcing the emotional experience of anger and the cognitive outlook of hostility. Social role theory proposes that females who exhibit non-normative traits, such as those in the lifestyle facet of psychopathy, may experience more anger because of societal backlash or stress. In contrast, the lack of a link between the affective facet and anger in females may reflect differences in socialization or coping strategies that buffer against anger expression, with females internalizing anger as anxiety or depression. Meta-analytic findings have suggested that there is a small positive association between the affective facet and anxiety and depression but did not test if sex moderated these effects (Batky et al., 2024). Further, prior research reports that psychopathy is related to emotional suppression (Walker et al., 2022) and emotional dysregulation (e.g., lack of impulse control in intense emotional states; Donahue et al., 2014). This suggests that mechanisms like emotional suppression and dysregulation differ by sex, though future research is needed.

### Aggressive behavior

The results show that all four psychopathy facets were related to both physical and verbal aggression, regardless of sex. Despite social expectations that may influence aggression (e.g., aggression being more socially accepted in males), psychopathic traits appear to directly drive aggressive behavior in both males and females, overriding these norms. This suggests that individuals with high levels of psychopathic traits may perceive aggression as justified, potentially because these traits align with traditionally masculine norms, such as dominance, fearlessness, and emotional detachments, which have been linked to aggression in prior research (e.g., Preston et al., 2018). This extends prior findings that only the affective and antisocial facets were linked to physical aggression (Thomson, 2020; Thomson et al., 2019b; Vitacco et al., 2005).

The findings contradict earlier research that reported a significant link between the affective facet and physical aggression only in females (Thomson et al., 2019b). The difference could be due to this sample, which included individuals with violent injuries, possibly normalizing aggressive behavior as a coping mechanism (Shulman et al., 2021; Widom, 1989). Unlike incarcerated participants, those in the community have more opportunities to engage in aggression, which may explain the strong connection between psychopathy and aggression in this study.

No sex differences were found in the relationship between psychopathy facets and verbal aggression, aligning with some research (e.g., Thomson et al., 2019b) but conflicting with others (Thomson, 2020). The lack of difference may again reflect participants’ history of violence. Interestingly, the affective and lifestyle facets were related to verbal aggression, differing from studies linking the interpersonal and antisocial facets to this outcome (Thomson et al., 2019b). However, recent online research supports our findings (Verona et al., 2023), suggesting that individuals with impulsive, emotionally shallow, and erratic traits may engage in verbal aggression without regard for consequences. Differences in psychopathy measures and sample characteristics could explain these inconsistencies, warranting future research.

Interestingly, the antisocial facet was related to physical aggression but not verbal aggression, anger, or hostility, despite its ties to past and present antisocial behavior, suggesting that lifestyle and affective facets play a more significant role in this sample.

### Practical implications

The findings have implications for clinical practice and intervention strategies, particularly in high-risk samples. While psychopathy is commonly used in clinical and justice systems to assess treatment needs and risk, this study suggests that the four facets are beneficial for addressing issues in high-risk community samples. This study demonstrates that violence interventions should integrate strategies for reducing psychopathic traits and that while males and females have common risk factors, there are sex differences, which should be accounted for in intervention and prevention efforts. Approaches tailored to each sex may enhance effectiveness. For males, interventions focused on emotion regulation and empathy enhancement may be effective, particularly for those with high affective traits. For females, addressing impulsivity and promoting responsible behavior could be key in managing anger and hostility for those with elevated lifestyle traits.

### Limitations

Some limitations should be considered when interpreting these results. First, compared to prior studies on sex differences in psychopathy and aggression, we did not test indirect aggression, which is higher among females (Thomson et al., 2019b; Thomson, 2020). However, we examined anger and hostility, which have been neglected by prior research. Second, this study did not test the function of aggression (i.e., reactive and proactive). Third, the study’s cross-sectional nature does not allow for causal inferences or temporal order, which would provide more actionable insights for interventions. Lastly, this study was focused on violently injured adults, a high-risk population; therefore, replication in more diverse populations is essential to ensure the findings are generalizable across varying demographics. Specifically, studies should examine cross-cultural variations in how psychopathy related to aggressiveness and whether these findings apply to other high-risk groups (e.g., juvenile delinquents).

## Conclusion

Psychopathy was related to verbal and physical aggression in males and females. Still, there were distinct sex patterns in anger and hostility, which is essential information for tailoring prevention and intervention strategies, ensuring that they address the unique needs of each sex effectively. Specifically, emotion regulation programs may be useful for males with high affective traits, while impulse control and problem-solving training may be useful for females with high lifestyle traits.

## Data Availability

The data presented in this study may be made available upon request from the corresponding author. The data are not publicly available due to the potential for personal identification of participants in the present sensitive population.

## Footnotes

^1^Separate analyses for each psychopathy facet revealed that all facets positively predicted aggression outcomes, confirming that the initial negative estimates for the interpersonal facet were due to suppression effects. The interactions remained consistent across these analyses.
